# Enhanced cutoff energies for direct and rescattered strong-field photoelectron emission of plasmonic nanoparticles

**DOI:** 10.1515/nanoph-2023-0120

**Published:** 2023-04-12

**Authors:** Erfan Saydanzad, Jeffrey Powell, Adam Summers, Seyyed Javad Robatjazi, Carlos Trallero-Herrero, Matthias F. Kling, Artem Rudenko, Uwe Thumm

**Affiliations:** J. R. Macdonald Laboratory, Department of Physics, Kansas State University, Manhattan, 66506, Kansas, USA; INRS, Énergie, Matériaux et Télécommunication, Varennes, J3X 1P7, Québec, Canada; Department of Physics, University of Connecticut, Storrs, 06269, CT, USA; SLAC, National Accelerator Laboratory, Menlo Park, 94025, CA, USA; Department of Applied Physics, Stanford University, Stanford, 94305, CA, USA

**Keywords:** electron source, nanoparticle, nanostructure, photonics, plasmonics, strong-field ionization

## Abstract

The efficient generation, accurate detection, and detailed physical tracking of energetic electrons are of applied interest for high harmonics generation, electron-impact spectroscopy, and femtosecond time-resolved scanning tunneling microscopy. We here investigate the generation of photoelectrons (PEs) by exposing plasmonic nanostructures to intense laser pulses in the infrared (IR) spectral regime and analyze the sensitivity of PE spectra to competing elementary interactions for direct and rescattered photoemission pathways. Specifically, we measured and numerically simulated emitted PE momentum distributions from prototypical spherical gold nanoparticles (NPs) with diameters between 5 and 70 nm generated by short laser pulses with peak intensities of 8.0 × 10^12^ and 1.2 × 10^13^ W/cm^2^, demonstrating the shaping of PE spectra by the Coulomb repulsion between PEs, accumulating residual charges on the NP, and induced plasmonic electric fields. Compared to well-understood rescattering PE cutoff energies for strong-field photoemission from gaseous atomic targets (10× the ponderomotive energy), our measured and simulated PE spectra reveal a dramatic cutoff-energy increase of two orders of magnitude with a significantly higher contribution from direct photoemission. Our findings indicate that direct PEs reach up to 93 % of the rescattered electron cutoff energy, in contrast to 20 % for gaseous atoms, suggesting a novel scheme for the development of compact tunable tabletop electron sources.

## 1 Introduction

The characterization of photoexcitation and -emission of plasmonic nanostructures is of basic research and applied interest for efficient harmonic up-conversion [1, 2], femtosecond time-resolved scanning tunneling microscopy and spectroscopy [3, 4], electron-impact spectroscopy [5, 6], and the development of compact electron sources [7]. We here show that prototypical plasmonic NPs exposed to intense IR-laser pulses emit PEs over a large kinetic energy range, owing to an intricate dynamical interplay of distinct electronic and photonic interactions. Extensively investigated during the past two decades [8, 9], metal NPs have remarkable optical properties that are primarily related to incident light in the IR to the visible frequency range enforcing the collective motion of conduction electrons. This light-driven excitation of localized surface-charge plasmons (LSP) controls the particles’ light absorption, reflection, and skin depths [10]. It results in a nanoplasmonic field near the NP surface that can greatly amplify the incident-laser electric field [11, 12]. The LSP resonance frequency of metal NPs can be tuned into resonance from IR to visible frequencies by variation of their shape, size, composition, and dielectric environment [8, 9, 13, 14]. This tunable enhanced light absorption and light scattering are key to powerful diagnostic methods, such as surface-enhanced Raman spectroscopy [15], time-resolved nanoplasmonic-field microscopy [12, 16], [17], [18], and biomedical and chemical sensing [19, 20]. The present single-pulse PE imaging investigation is expected to promote future two-pulse pump-probe experimental schemes for the spatiotemporal imaging of induced-plasmonic-field distributions near the surface of metal nanoshells that have recently been proposed in classical [21] and quantum-mechanical [12, 18, 22] numerical simulations.

In this work, we have employed 2-dimensional velocity-map-imaging (VMI) spectroscopy to investigate strong-field electron emission from metal NPs. VMI spectroscopy provides projections of PE momentum distributions onto the plane of a 2-dimensional PE detector. It is established as a powerful technique for studying intense-light interactions with atoms and molecules [23–25]. Through the last decade, this technique was applied to study strong-field photoemission from isolated NPs by intense linearly polarized laser pulses [26–28]. During strong-field emission from atoms and molecules [29], PEs can gain a significant amount of energy while propagating in the oscillating laser electric field. PEs that are “directly” emitted from gaseous atomic targets by linearly polarized laser pulses (without being driven by the external light field to return to the residual ion) gain up to 2 *U*_p_(*I*_0_) in kinetic energy, while PEs that are accelerated back to the residual ion by the laser electric field to “rescatter” elastically accumulate up to 10 *U*_p_(*I*_0_) [30–33]. The ponderomotive energy *U*_p_(*I*_0_) = *I*_0_/(4*ω*^2^) is the cycle-averaged quiver energy of a free electron in a laser field of frequency *ω* and peak intensity *I*_0_. Unless indicated otherwise, we use atomic units throughout this work. For strong-field PE emission and rescattering from solids [34–40] and nanostructures, such as nanotips [3, 41], [42], [43], [44], [45], [46], isolated clusters [47–51], and dielectric NPs [27, 28, 52], cutoff energies in directly emitted and rescattered photoemission from dielectric NPs were found to be approximately 2 *η*^2^*U*_p_(*I*_0_) and 10 *η*^2^*U*_p_(*I*_0_), respectively [27, 53]. Compared to atomic targets, these limiting PE energies are enhanced by the square of the near-field plasmonic-enhancement factor *η*. In this work, we (i) measured and numerically modeled VMI spectra resulting from the strong-field PE emission from metal NPs by intense IR-laser pulses and (ii) validated a recent extension [17] of the three-step-model for atomic strong-field ionization [54] to metal NPs. Owing to plasmonic-near-field enhancement of the incident-laser electric field and PE correlation, we found measured and calculated cutoff energies for metal NPs that exceed typical cutoff energies from gaseous atoms and dielectric NPs by two and one order of magnitude, respectively. Interestingly, the cutoff energy for direct electron emission from metal NPs reaches up to 93 % of the corresponding value for rescattered PEs electrons, dramatically exceeding the well-known proportion of 20 %, discussed earlier for gaseous atoms and dielectric NPs [27, 53].

## 2 Methods

### 2.1 Experimental setup

**Figure 1:**
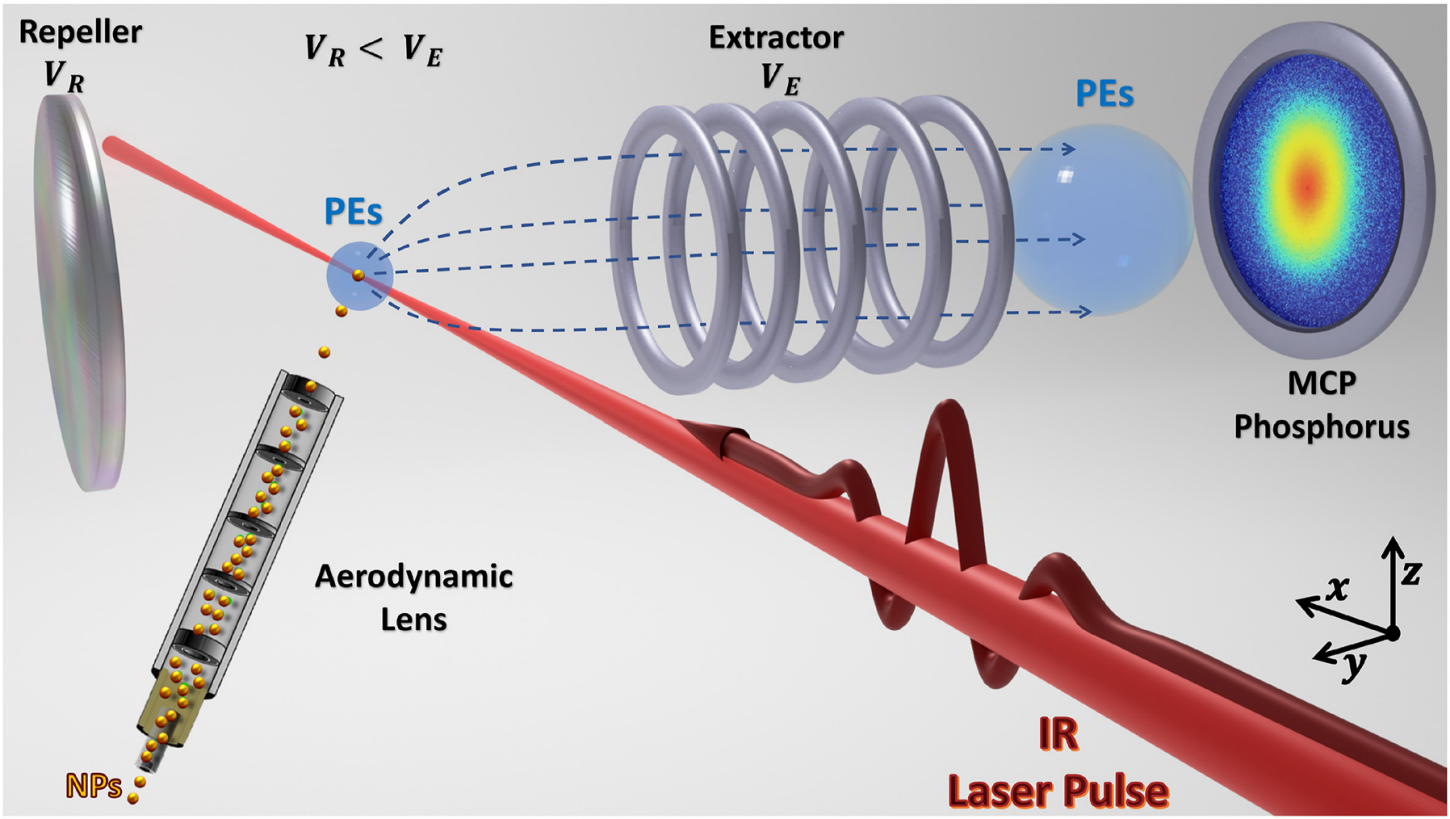
Schematic of the velocity-map-imaging spectrometer coupled to the nanoparticle source. The dilute beam of isolated gas-phase nanoparticles is injected into vacuum and focused by an aerodynamic lens to intersect 800 nm, 25 fs, 10 kHz-repetition-rate linearly polarized laser pulses. Emitted electrons are focused onto the microchannel plate (MCP)/phosphor assembly. *V*_R_ and *V*_E_ are the respective voltages on the PE repeller and extractor plates needed to guide photoelectrons to the MCP phosphorus detector. The MCP is coupled to a phosphor screen, of which a camera records the spatial distribution of photoelectron hits for every laser shot.

The laser system and VMI electron detection apparatus at the James R. Macdonald Laboratory at Kansas State University are described in more detail in [26, 55]. Briefly, the experiments used a Ti:Sapphire-based chirped pulse amplification (CPA) system generating 25 fs pulses FWHIM (10 optical cycles), and central angular frequency *ω* = 2.415 PHz (corresponding to a central wavelength *λ* = 780 nm). The laser pulse intersects the stream of isolated single NPs with diameters of 5, 30, or 70 nm that are injected by aerodynamic lens focusing [28, 55], [56], [57], [58]. As shown in the sketch of the experimental setup in Figure 1, PEs are projected onto the detector by the static electric field between the repeller and extractor. This allows the recording of the 2D projection of the PE momentum distributions as VMI maps. PE spectra were captured in a thick-lens, high-energy VMI spectrometer [59] capable of gathering up to 350 eV electron energy. The NPs were purchased from Cytodiagnostics [60]. The NP samples were custom synthesized, characterized for monodispersity (typical polydispersity index < 0.1) and sphericity (>95 %) to ensure sufficient reproducibility between interactions, and extensively purified to remove any source of contamination. We carefully chose the initial NP concentration to avoid the formation of clusters in the NP beam [61].

### 2.2 Laser-intensity characterization

The peak laser intensity was determined by analyzing the above-threshold-ionization (ATI) PE energy distribution from gaseous Xe atoms with the VMI spectrometer described above and for the same laser parameters we selected for the strong-field-ionization studies reported in this work. To determine the absolute value of the intensity, the ponderomotive shift of the Xe ATI comb was measured as a function of the input-laser-pulse energy. From this shift, we deduced the ponderomotive energy, *U*_P_, for a given pulse energy. Since *U*_p_ is proportional to the peak laser intensity *I*_0_, the latter could be directly determined from this measurement. We determined the values of the intensities used in this work as *I*_0_ = 8.0 × 10^12^ W/cm^2^ and 1.5 *I*_0_ and estimated the accuracy of the intensity calibration to be better than 15 % (see Ref. [55, 62] for details).

### 2.3 Theoretical model

We numerically investigated PE emission from metallic NPs by IR-laser pulses with a Gaussian temporal profile. Propagating along the *x* axis and linearly polarized along the *z* axis, their electric field is given by

(1) $$\begin{array}{cc} {\vec{E}}_{\text{inc}}\left(\vec{r},t\right) & =\sqrt{I_{0}}\exp\left[-2\ln2\frac{{\left(t-x/c\right)}^{2}}{\tau^{2}}\right]\\ & \;\times \exp\left[-\text{i}\omega \left(t-x/c\right)+\text{i}\varphi \right]\;{\hat{e}}_{z}, \end{array}$$

where *τ* is the pulse length at full-width-half-intensity maximum (FWHIM), *ω* the pulse’s central frequency, *φ* the carrier-envelope phase, and *c* the speed of light in vacuum (Figure 1). During the laser – NP interaction, LSPs are excited and induce an inhomogeneous plasmonic field near the NP surface. At the same time, and most significantly at the LSP resonance frequency [63, 64], electrons are excited to electronic states above the Fermi level. Sufficiently high laser intensities generate multiply ionized NPs [26, 56].

The incident laser pulse induces a transient dipole in the NP. Within the electric-dipole approximation, the corresponding transient induced plasmonic-dipole moment, ${\vec{P}}_{\text{pl}}\left(t\right)=\epsilon_{0}\alpha_{\text{Mie}}\left(\omega \right){\vec{E}}_{\text{inc}}\left(\vec{r},t\right)$ , generates the plasmonic electric field [65]

(2) $$\begin{array}{ll} {\vec{E}}_{\text{pl}}\left(\vec{r},t\right) & =\frac{{\text{e}}^{\text{i}\mathrm{kr}}}{r}\left\{k^{2}\left[{\hat{e}}_{r}\times {\vec{P}}_{\text{pl}}\left(t\right)\right]\times {\hat{e}}_{r}\right.\\ & \;\left.+\left[3{\hat{e}}_{r}\left[{\hat{e}}_{r}\cdot {\vec{P}}_{\text{pl}}\left(t\right)\right]-{\vec{P}}_{\text{pl}}\left(t\right)\right]\left(\frac{1}{r^{2}}-\frac{\text{i}k}{r}\right)\right\}, \end{array}$$

where *k* = 2*π*/*λ* = *ω*/*c*. We calculate the complex NP polarizability, *α*_Mie_(*ω*), within Mie theory [66], following Ref. [67], which restricts the applicability of Eq. (2) to size parameters *ka* ⪅ 0.6 for nanospheres of radius *a* [68].

We describe strong-field ionization from metal NPs by extending the semi-classical three-step model (also known as “simple-man model”) for atomic strong-field ionization to metal NPs [17]. Our extended three-step model consists of: (1) electron release based on quantum-mechanical tunneling, (2) PE propagation from the NP surface to the detector by sampling over classical trajectories, and (3) PE rescattering and recombination at the NP surface. In comparison with gaseous atomic targets, each of these steps is significantly more intricate for metal NPs, due to their more complex electronic structure, the added morphological structure, and the emission of a much larger number of electrons, emphasizing the effects of PE – PE correlation, residual charges, and PE – nanoplasmonic-field interactions.

We represent the NPs’ static electronic structure in terms of the surface-potential step *V*_0_ = *ɛ*_*F*_ + *φ* with the work function *φ* = 5.1 eV and Fermi energy *ɛ*_*F*_ = 8.0 eV for bulk gold [69]. Our dynamical numerical simulation divides the NP surface into small surface elements. During successive small time intervals, the surface elements are modeled as spherical square-well potentials. Bound PEs close to the NP surface tunnel out along the radial component of the total electric field at the NP surface, $\vec{F}\cdot {\hat{e}}_{r}$ , where $\vec{F}={\vec{E}}_{\text{inc}}+{\vec{E}}_{\text{pl}}+{\vec{F}}_{\text{res}}$ . The residual-charge field ${\vec{F}}_{\text{res}}$ results from the accumulation of positive residual charge on the NP during electron emission in preceding time intervals. We account for strong-field electron release from the NP by employing modified [17] Fowler–Nordheim tunneling rates [70, 71]. Subsequently, we Monte Carlo sample over the initial phase-space distribution of released electrons and solve Newton’s equations of motion for the PE propagation outside the NP in the presence of all electric fields, $\vec{F}+{\vec{F}}_{e-e}$ , where ${\vec{F}}_{e-e}$ is the repulsive Coulomb electric field between PEs. In each laser half-cycle the direction of the incident-laser electric field changes, such that emitted PEs can be driven back toward the NP and either rescatter from or recombine at the NP surface. For 5, 30, and 70 nm diameter gold nanospheres, we include and numerically evaluate the effects of PE repulsion, residual positive charges on the NP, PE recollisions and recombinations at the NP surface, and nanoplasmonic enhancement of the incident-laser-pulse electric field. More details about this numerical model are given in the Supplementary Information (SI) and in Ref. [17].

In our numerical applications in Section 3, we distinguish and compare specular and diffusive PE rescattering at the NP surface. For diffusive rescattering, we uniformly randomize the polar and azimuthal scattering angles relative to the surface normal at the impact site on the NP surface, modeling rescattering in all accessible directions with equal probability.

## 3 Experimental and simulation results

### 3.1 Influence of nanoplasmonic field, rescattering, residual-charge interactions, and photoelectron correlation

VMI spectra are sensitive to all PE interactions included in our simulation. In order to track the effects of different electronic interactions on the propagation and rescattering of released PEs, we leave the modeling of the tunneling release of electrons at the NP surface unchanged when selectively switching off individual PE interactions (for identical laser-pulse parameters), assuming for all calculated VMI maps identical tunneling-ionization rates (Eq. (S1.5) in the Supplementary Information) The comparison of simulations in which we selectively include and exclude specific PE interactions during the PE propagation and rescattering, allows us to quantify their specific effects on VMI maps.

**Figure 2:**
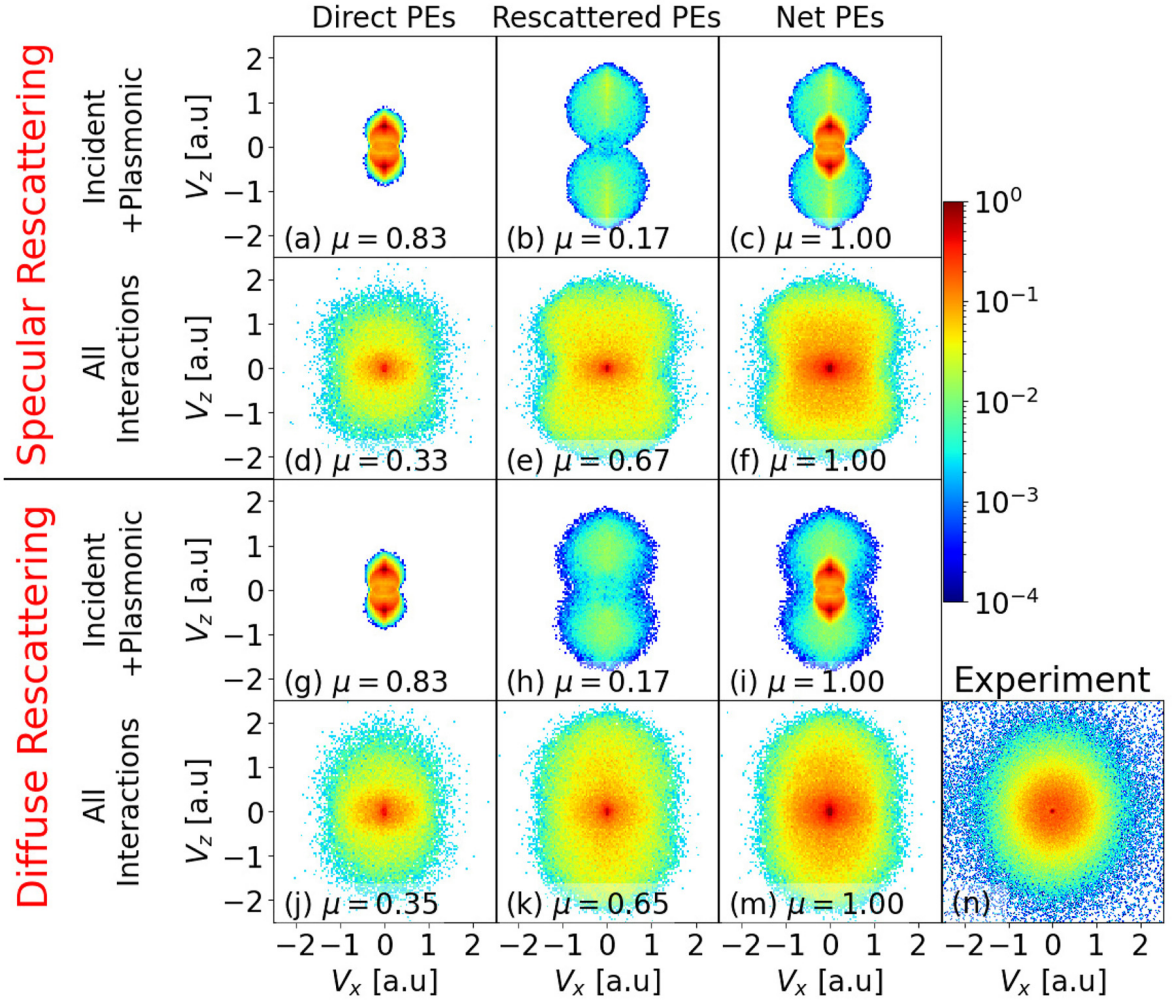
(a–m) Photoelectron VMI spectra simulated for 30 nm diameter gold nanospheres for direct (first column), rescattered (second column), and all (denoted as “Net”, third column) photoelectrons, including either specular (first and second row) or diffuse rescattering (third and fourth row) for incident 780 nm laser pulses with a pulse length of 25 fs (FWHIM) and 8.0 × 10^12^ W/cm^2^ peak intensity. *μ* designates the integrated yield, normalized to the simulated net PE yield. First and third row: simulations where only the incident-laser and plasmon fields ( ${\vec{E}}_{\text{inc}}$ and ${\vec{E}}_{\text{pl}}$ ) are included. Second and fourth row: VMI spectra including all the interactions, ${\vec{E}}_{\text{inc}}$ , ${\vec{E}}_{\text{pl}}$ , photoelectron interactions with residual positive charges $\left({\vec{F}}_{\text{res}}\right)$ , and repulsive photoelectron Coulomb interactions $\left({\vec{F}}_{e-e}\right)$ . (n) Corresponding measured VMI spectrum.

Figure 2 shows simulated VMI spectra compared to experimental results for gold nanospheres with 30 nm diameter for the experimental setup depicted in Figure 1. The VMI spectra are projections of the PE momentum distribution on the *x*–*z* plane of the MCP detector and show the projected PE yields as functions of the PE asymptotic velocities, *v*_*x*_ and *v*_*z*_, along the laser-propagation and -polarization directions. The first, second, and third column in Figure 2 include, respectively, VMI spectra of direct PEs, rescattered PEs, and the net PE yield for either specular (first and second row) or diffuse rescattering (third and fourth row, cf., Sec. (S3) in the SI). The first and third row show simulations in which only the incident-laser and plasmon fields ( ${\vec{E}}_{\text{inc}}$ and ${\vec{E}}_{\text{pl}}$ ) are considered. The VMI spectra in the second and fourth row include all forces: ${\vec{E}}_{\text{inc}}$ , ${\vec{E}}_{\text{pl}}$ , PE interactions with residual positive charges $\left({\vec{F}}_{\text{res}}\right)$ , and repulsive PE Coulomb interactions $\left({\vec{F}}_{e-e}\right)$ . Figure 2(n) is our measured VMI map for the same laser and NP parameters.

To allow for a quantitative comparison of direct and rescattered PE yields, we normalized the yields in each row to the corresponding net PE yield in the third column and display the normalized integrated yield *μ* in each graph of Figure 2. We calculated *μ* as the *v*_*x*_- and *v*_*z*_-integrated yields from the simulated VMI maps in each row, divided by the corresponding integrated yield of the VMI maps in the third column. The comparison of the VMI spectra in Figure 2 allows us to assess the influence of the distinct PE interactions on VMI spectra, as we discuss next.

#### 3.1.1 Plasmonic-field interactions

The simulated VMI spectra in the first and third row of Figure 2 are calculated under the assumption that released electrons solely interact with the incident-laser and induced plasmonic fields while propagating to the detector. These PE distributions are aligned with the laser-polarization direction and have a dipole-like appearance, owing to the dipole character transferred from the induced plasmonic field and tunneling ionization.

The comparison of Figure 2(a) and (b) with Figure 2(c) for specular rescattering and Figure 2(g) and (h) with Figure 2(i) for diffuse rescattering reveals that directly emitted PEs dominate the low-energy part of the photoemission spectra. Rescattered PEs, in contrast, can gain additional energy from the laser and induced plasmonic fields and establish the higher-energy part of the PE spectrum. Rescattering boosting PE energies is a well-understood phenomenon in strong-field ionization. For gaseous atomic targets, elastically rescattered PEs reach kinetic energies up to 10 *U*_p_(*I*_0_) [30–33] and larger energies occur for dielectric NPs (SiO_2_) [26, 27, 56]. By comparing the yield factors *μ* in the first and second row, we find that approximately 83 % of the detected PEs is directly emitted, while 17 % have rescattered at the NP surface at least once.

#### 3.1.2 All interactions effect

The second (specular rescattering) and forth (diffuse rescattering) row of Figure 2 show simulated VMI spectra including all PE interactions, i.e., ${\vec{E}}_{\text{inc}}$ (1), ${\vec{E}}_{\text{pl}}$ (2), ${\vec{F}}_{e-e}$ [Eq. (S2.7) in the SI], and ${\vec{F}}_{\text{res}}$ [Eq. (S1.4) in the SI]. The inclusion of the long-range Coulomb attraction of accumulating positive residual charges decelerates both direct and rescattered PEs, increasing the number of PEs that recombine with and rescatter off the NP. The addition of ${\vec{F}}_{e-e}$ opposes the residual-charge interaction by introducing Coulomb repulsion into the system of released electrons, accelerating a large fraction of PEs to significantly higher final (detectable) kinetic energies.

As noted above, in the absence of PE–PE interactions and diffuse rescattering, the linearly polarized incident-laser and induced plasmonic electric field imprint their dipole character on the VMI spectra. The inclusion of PE–PE interactions and diffuse rescattering partially removes the dipolar emission character and results in more isotropic VMI spectra [17]. For metal NPs, attractive residual-charge interactions are thus much less influential than PE–PE interactions in shaping PE momentum distributions and determining PE cutoff energies.

Comparing the VMI spectra in rows one and two of Figure 2 for specular and in rows three and four for diffuse rescattering, we notice that the combined effect of ${\vec{F}}_{e-e}$ and ${\vec{F}}_{\text{res}}$ considerably increases the final energy of directly emitted electrons, while decreasing the direct-emission yield from 83 % to 33 % (specular rescattering) and 35 % (diffuse rescattering). On average, directly emitted PEs are slower than rescattered PEs and thus spend more time near the NP. They are therefore (i) more likely to recombine with the NP, reducing the direct PE yield, and (ii) experience stronger PE–PE Coulomb repulsion, leading to higher acceleration and larger final kinetic energies. Due to influential PE–PE interactions, direct photoemission reaches a cutoff energy of 121 *U*_p_(*I*_0_) for 30 nm diameter NPs. This is 85 % the cutoff energy for rescattered PEs [cf., Figure 2(d) and (j)]. Thus, PE–PE interactions significantly contribute to the high-energy part of the PE spectra, even for direct emission, resulting in cutoff energies significantly larger than the known 2 *U*_P_(*I*_0_) limit of atomic targets [31] and even the 2 *η*^2^*U*_P_(*I*_0_) cutoff energy of dielectric NPs [27]. The increase of the PE cutoff energies due to rescattering, and as compared to direct emission, is less pronounced for metal NPs than for gaseous atomic targets and dielectric NPs.

Figure 2(n) shows our experimental VMI spectra. With regard to yield, cutoff energy, and isotropic shape of the PE momentum distribution, Figure 2(m) (including all interactions and with diffuse rescattering) is our most comprehensive simulation result and matches the experiment well. The VMI spectra in Figure 2 clearly show that all PE interactions are relevant for shaping the PE angular distribution in the measured VMI spectrum in Figure 2(n).

### 3.2 Influence of nanoparticle size and laser intensity

**Figure 3:**
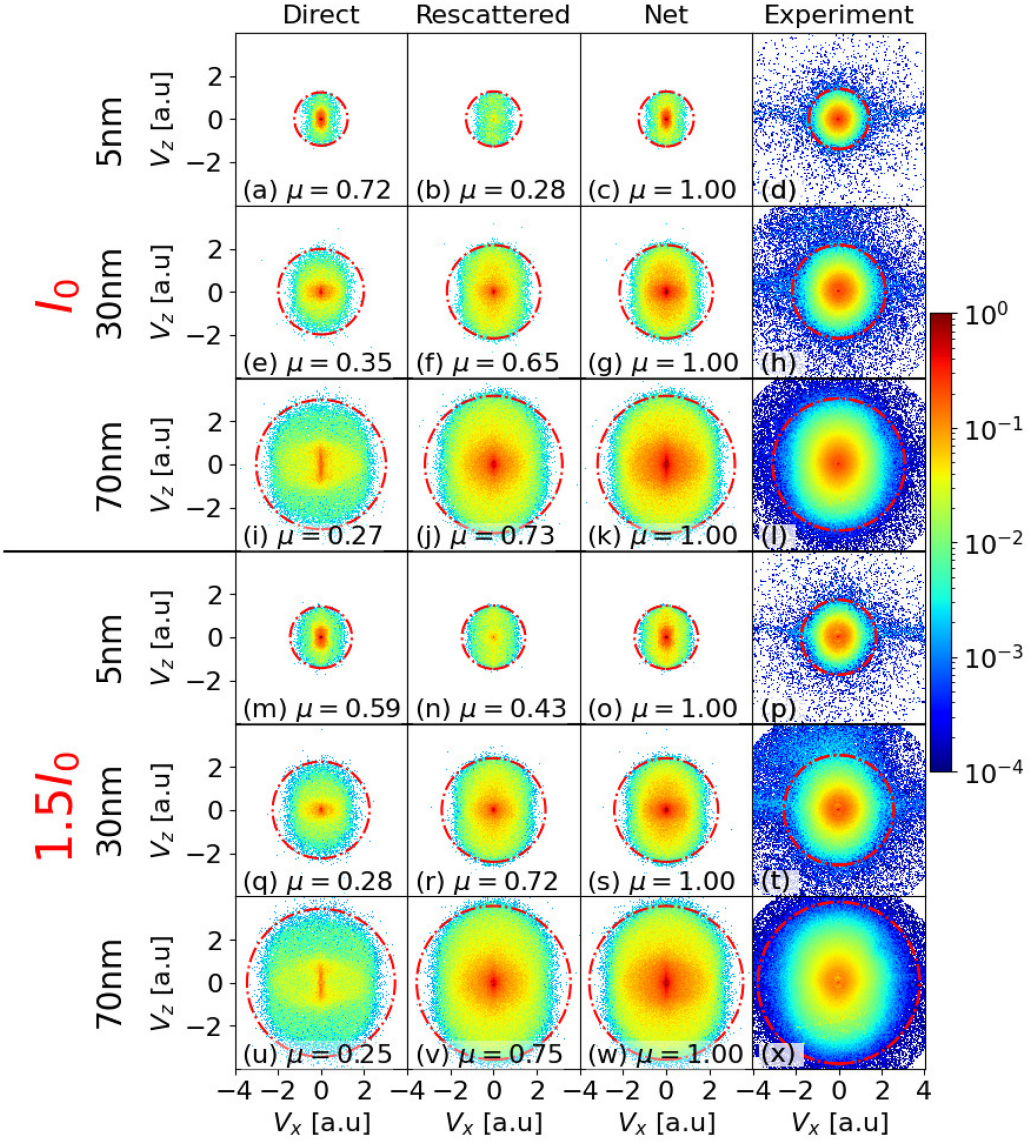
Comparison of simulated direct (first column), rescattered (second column) and net (i.e., including direct and rescattered yields, third column) photoelectron VMI spectra with experimental (forth column) VMI spectra for gold nanospheres with 5, 30, and 70 nm diameter and laser peak intensities of *I*_0_ = 8.0 × 10^12^ W/cm^2^ (first – third row) and 1.5 *I*_0_ (forth – sixth row). The laser-pulse length and wavelength are 25 fs and 780 nm. Red dashed circles in (a–x) indicate simulated and experimental photoelectron cutoff energies. *μ* is the integrated photoelectron yield normalized to the integrated net yields in third column.

Figure 3 shows simulated and experimental VMI spectra for gold nanospheres with diameters of 5, 30, and 70 nm. The first, second, and third column are simulated VMI spectra for, respectively, the direct, rescattered, and net PEs yield for peak laser intensities *I*_0_ (first, second, and third row) and 1.5 *I*_0_ (fourth, fifth, and sixth row). Experimental results corresponding to the simulations in the third column are shown in the fourth column. The VMI spectra in Figure 3 are (slightly) elongated along the laser-polarization direction, with PE cutoff energies that increase with NP size. As discussed in Section 3.1, isotropic VMI spectra are promoted by PE–PE interactions and diffuse PE rescattering from the NP surface, while incident-laser and induced plasmonic-field interactions tend to imprint a dipolar shape. The *detected* number of the PEs per laser short for the experimental data shown in Figure 3 varies from 140 for 5 nm diameter NPs at the lower peak laser intensity (*I*_0_) to 600 for 70 nm NPs at 1.5 *I*_0_. However, as discussed in detail in Ref. [55], these numbers do not directly reflect the number of PEs that *hit* the detector due to the PE energy-dependent detector saturation in our experiment. The saturation effect is most prominent in the central detection area, where low-energy electrons (which dominate the total PE yield) hit the MCP.

To allow for a quantitative comparison of direct and rescattered PE yields, we normalized the direct and rescattered PE yields in each row to the corresponding net PE yield in the third column and displayed the normalized integrated yield *μ*(*a*, *I*_0_) in each graph. *μ* reveals that the yield of direct PEs decreases as a function of the NP size and intensity, being more sensitive to the size. This observation is compatible with PEs having a higher probability to rescatter off larger NPs. In addition, increasing laser intensity leads to a stronger radial attractive force, due to an increase in the number of residual charges on the NP surface, leading to more PE rescattering events. The direct and rescattered PE yield can be controlled by the intensity of the laser pulse and size of the NP. The measured and simulated VMI maps also reveal a large increase in the direct and rescattered PE cutoff energy with the laser peak intensity and NP size. We quantify this laser-intensity and NP-size-dependent effect in the following subsection.

### 3.3 Angle-integrated photoelectron yields and cutoff energies

**Figure 4:**
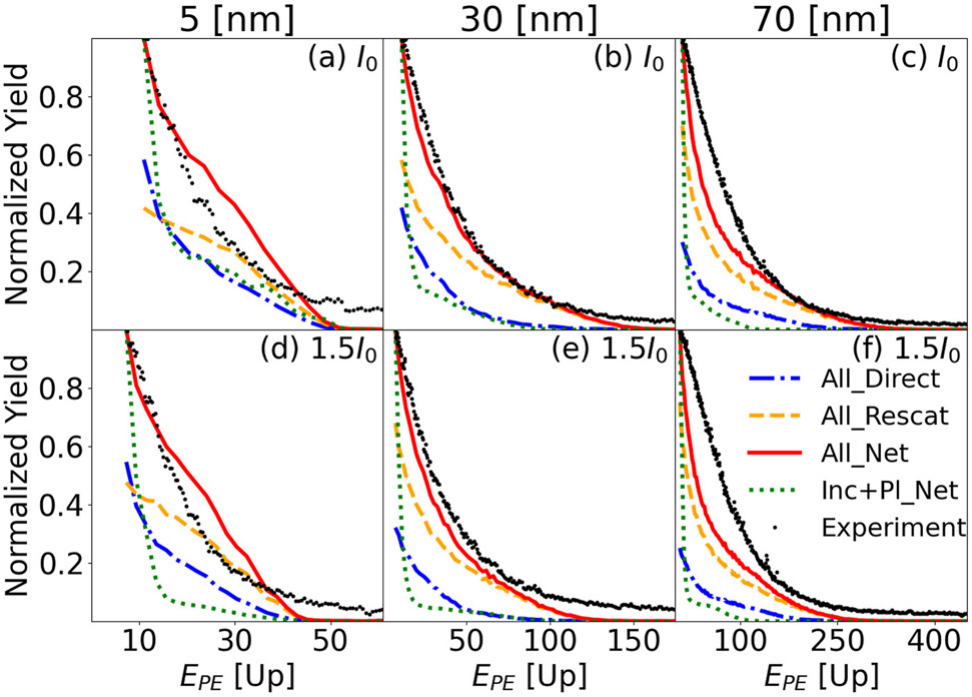
Photoemission yields as functions of the photoelectron kinetic energy for gold nanospheres with (a) and (d) 5, (b) and (e) 30, and (c) and (f) 70 nm diameter and laser peak intensities of (a–c) *I*_0_ = 8.0 × 10^12^ W/cm^2^ and (d–f) 1.5 *I*_0_. The laser-pulse length and wavelength are 25  fs and 780 nm. Simulated photoelectron yields including all interactions are shown for directly emitted (“All_Direct”), rescattered (“All_Rescat”), and net (“All_Net”, i.e., direct and rescattered) photoelectrons. Simulations only including incident- and plasmonic-field interactions are denoted as “Inc + Pl_Net”. Black dots show experimental yields.

Figure 4 shows simulations corresponding to the VMI spectra in Figure 3. It includes (i) all interactions for the direct PE yield (denoted as “All_Direct”), (ii) all interactions for the rescattered PE yield (“All_Rescat”), and (iii) all interactions for the net PE yield (“All_Net”). In addition, Figure 4 displays (iv) simulations only including incident- and plasmonic-field interactions for the net PE yield (denoted as “Inc + Pl_Net”) and (v) integrated experimental yields as a function of the PE kinetic energy. Due to the detector saturation at the center of the MCP phosphor detector (Figure 1), the experimental yields for kinetic energies below approximately 8 eV (corresponding to PE velocities below 0.8 a.u.) are not accurate. To be able to compare experimental integrated yields to one another and to the simulation results, we have removed the low energy part of the integrated yields from both, experimental and simulated data.

The overall agreement between experimental and simulated integrated PE yields in Figure 4 is not perfect for several reasons. With regard to the simulation, an important uncertainty derives from our implementation of approximate modified Fowler–Nordheim tunneling rates. With regard to the experiment, the above-mentioned detector saturation decreases the reliability of the low-energy portion of our spectra. While the low-energy portion of the simulation data was truncated to allow for a better comparison with the experiment, the detection uncertainty due to saturation is not completely removed and tends to affect predominantly our measurements for the largest NP size (70 nm diameter) and the higher laser peak intensity (1.5 *I*_0_), due to larger numbers of emitted PEs per laser shot. This is consistent with the agreement between simulation and measurement being better for 30 nm NPs at the lower peak intensity (*I*_0_) in Figure 4 than for 70 nm NPs in Figure 4(c) and at the higher laser intensity of 1.5 *I*_0_ in Figure 4(d)−(f). However, in view of hardly avoidable inaccuracies in the detailed modeling of this complex interaction scenario and NP-size- and laser-intensity-dependent experimental background noise, we cannot exclude that the exceptionally good match between experimental simulated results shown in 4(b) compared to the other graphs in this figure is serendipitous.

Integration of the VMI-projected PE momentum distributions *y*(*v*_*x*_, *v*_*z*_) in Figure 3(a)–(x) over the PE detection angle *ϕ* in the VMI plane results in PE yields

(3) $$Y\left(E_{\text{PE}}\right)=\int d\phi \;y\left(\sqrt{2E_{\text{PE}}}\cos\phi ,\sqrt{2E_{\text{PE}}}\cos\phi \right)$$

as functions of the PE energy, $E_{\text{PE}}=\left(v_{x}^{2}+v_{z}^{2}\right)/2$ . The yields *Y*(*E*_PE_) shown in Figure 4(a)–(f) are normalized individually to their maxima, except for the simulations labeled “Direct_Net” and “Rescat_Net”, which are normalized to the maxima of the “All_Net” simulation results.

For simulated yields, we define the PE cutoff energy *E*_cutoff_ as the energy up to which 99.5 % of the net PE yield has accumulated,

(4) $$\frac{\overset{E_{\text{cutoff}}}{\underset{0}{\int }}\;\text{d}E_{\text{PE}}Y\left(E_{\text{PE}}\right)}{\overset{\infty }{\underset{0}{\int }}\;\text{d}E_{\text{PE}}Y\left(E_{\text{PE}}\right)}=99.5\;\%.$$

The experimental cutoff energy was extracted from the experimental VMI maps as described in [26, 55], for which the upper energy boundaries of the full 3D momentum sphere and the 2D projection are identical. The radial distribution of these projections along the polarization direction accurately determines the maximum PE energy.

**Figure 5:**
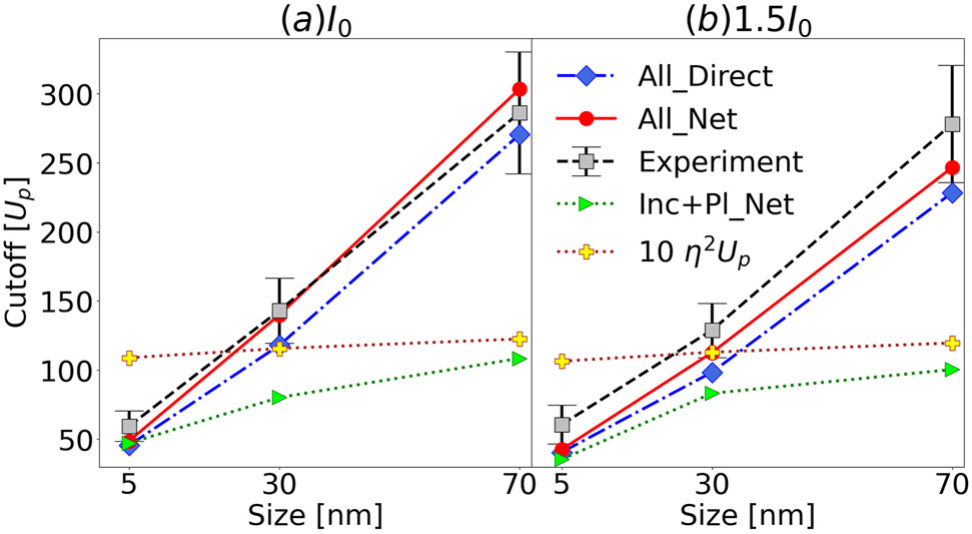
Comparison of simulated and experimental photoelectron cutoff energies scaled by the incident-laser ponderomotive energy *U*_p_(*I*_0_) for 5, 30, and 70 nm diameter gold nanospheres and laser peak intensities of (a) *I*_0_ = 8.0 × 10^12^ W/cm^2^ and (b) 1.5 *I*_0_. The laser-pulse length and wavelength are the same as in Figure 4. Simulated cutoff energies including all interactions for, respectively, direct (“All_Direct”) and net (“All_Net”, i.e., direct and rescattered) photoemission. Simulations only including incident- and plasmonic-field interactions are denoted as “Inc + Pl_Net”. Yellow “plus” markers show atomic cutoff energies, 10 *U*_p_, scaled by the plasmonic intensity enhancement *η*^2^.

The PE cutoff energies, shown as red dashed circles in Figure 3 in Section 3.2, increase with the NP size and peak laser intensity. Figure 5(a) and (b) display cutoff energies as a function of NP size for peak intensities of *I*_0_ = 8.0 × 10^12^ W/cm^2^ and 1.5 *I*_0_, in units of the incident-laser ponderomotive energies *U*_p_(*I*_0_) and *U*_p_(1.5 *I*_0_), respectively. Blue diamonds and red circles show, respectively, simulated cutoff energies (including all interactions) for the direct (denoted as “All_Direct”) and net (“All_Net”) PE yields. Gray squares with error bars are experimental cutoff energies (“Experiment”). Simulation results including all interactions for rescattered PEs are not shown, because they coincide with the “All_Net” yield. For gaseous atomic targets, the cutoff energy is equal to 10 *U*_p_ [30–33]. Cutoff energies obtained by scaling this well-known expression by the plasmonic intensity enhancement of the incident-laser pulse, *η*^2^, are shown as yellow “plus” markers. As expected, they tend to merge with the cutoff energies computed while only including incident-laser-pulse and plasmonic-field interactions (represented as green triangles). We calculated the applied value for *η* within Mie theory at the poles (relative to the laser-polarization direction) of the NPs [66–68]. In contrast to the 10 *η*^2^*U*_p_ scaling, the comparison of Figure 5(a) and (b), shows that our theoretical cutoff energies predict intensity-dependent changes that become more pronounced for larger NPs. Within the experimental error this theoretical prediction is compatible with our experimental results.

Based on the discussion in Section 3.1 of different PE interactions and their influence on VMI maps, we investigated two plausible causes for the numerically predicted increase of the PE yield and cutoff energy with the NP size. First, the lowering and narrowing of the surface-potential barrier by the more significant nanoplasmonic-field enhancement near larger NPs [12, 18, 21, 68] promotes strong-field tunneling ionization. However, this not only tends to augment the measured PE yield. Since PEs gain more energy in a more strongly enhanced field, it also entails higher cutoff energies for larger NPs. Second, as the NP size increases, a larger surface area becomes available from where more electrons are emitted, increasing the PE yield. The cutoff energy rises with the PE yield due to the increased repulsive Coulomb energy between PEs upon their release from the NP surface. In principle, a third cause for larger yields and cutoff energies can be laser-pulse-propagation effects inside the NP that result in higher local-field enhancements for larger NPs [56]. However, for the NP sizes investigated here, we did not find this effect to be relevant.

As discussed in Section 3.1, the consequences of residual-charge interactions and PE–PE interactions oppose each other. While attractive residual-charge – PE interactions reduce both, PE yields and cutoff energies, PE Coulomb repulsion increases them. A detailed numerical comparison of these competing interactions is shown in Figure 3 of Ref. [17]. Our numerical results indicate that PE Coulomb repulsion overcompensates residual-charge – PE interactions with regard to the cutoff energy, leading to an overall cutoff-energy increase, especially for larger NPs.

The green triangles (denoted as “Inc + Pl_Net”) in Figure 5 are cutoff energies calculated under the assumption that released electrons solely interact with the incident-laser and induced plasmonic field while propagating to the detector. In Sec. (S4) of the SI, we derive a closed-form analytical heuristic expression for the cutoff energy in direct photoemission, based on a simplified central-field approximation of residual-charge interactions and PE correlation. By comparing Eq. (S4.12) in the Sec. (S4) with the known respective 2 *U*_p_ and 10 *U*_P_ limits for direct and rescattered emission for atomic strong-field ionization, we infer the cutoff energy for rescattered PEs,

(5) $$E_{\text{cutoff}}^{R}=10\;U_{\text{p}}{\left[\eta_{\text{eff}}^{R}\left(a,I_{0}\right)+\omega t_{f}\eta_{\text{C}}^{R}\left(a,I_{0}\right)\right]}^{2},$$

where $\eta_{\text{eff}}^{R}\left(a,I_{0}\right)$ models, on average, the effect of plasmonic-field enhancement on rescattered PEs (indicated by the superscript “R”), while taking all PE interactions into account. *t*_*f*_ designates the effective interaction time (determined at numerical convergence). We introduce the effective Coulomb interaction factor, $\eta_{\text{C}}^{R}\left(a,I_{0}\right)=\eta_{e-e}^{R}\left(a,I_{0}\right)-\eta_{\text{res}}^{R}\left(a,I_{0}\right)$ , in analogy to the plasmonic-field-enhancement factor *η*. In the central-field approximation, $\eta_{e-e}^{R}\left(a,I_{0}\right)$ and $\eta_{\text{res}}^{R}\left(a,I_{0}\right)$ represent PE–PE repulsion and the decelerating effect of residual-charge interactions, respectively. Note that direct PEs, on average, are more strongly affected by repulsive PE–PE Coulomb interactions and plasmonic-field enhancement than rescattered electrons, as mentioned earlier. For the NP and laser parameters we considered, this leads to comparable cutoff energies for direct emission and rescattering. $\eta_{\text{C}}^{R}\left(a,I_{0}\right)$ is a measure for the magnitude of the effective counteracting attractive (decelerating) residual-charge and repulsive (accelerating) PE–PE interactions. For the present work $\eta_{\text{C}}^{R}\left(a,I_{0}\right)>0$ , indicating the dominance of PE repulsion over residual-charge attraction in determining the cutoff energy.

The heuristic Eq. (5) qualitatively explains all experimental results in Figure 5. For very small $\eta_{\text{C}}^{R}\left(a,I_{0}\right)$ and plasmonic-field enhancement, Eq. (5) approaches the familiar 10 *U*_p_(*I*_0_) scaling for rescattering ionization of gaseous atomic targets, as expected. This condition is satisfied for dielectric NPs for appropriate particles sizes and laser intensities. Including field enhancement simply in terms of a multiplicative factor, *η*^2^, i.e., modeling cutoff energies as 10 *η*^2^*U*_p_(*I*_0_), fails to reproduce the cutoff energies we measured and simulated for metal NPs when plasmonic-field enhancement and PE Coulomb interactions are relevant. If $\eta_{\text{C}}^{R}\left(a,I_{0}\right)$ is negligible, the cutoff energy, $10\;{\left[\eta_{\text{eff}}^{R}\left(a,I_{0}\right)\right]}^{2}U_{\text{p}}\left(I_{0}\right)$ , is smaller than 10 *η*^2^*U*_p_(*I*_0_), since $\eta_{\text{eff}}^{R}\left(a,I_{0}\right)<\eta$ , (see Sec. (S4) in the SI). For $\eta_{\text{C}}^{R}\left(a,I_{0}\right)>0$ , PE correlation dominates the residual-charge deceleration, and cutoff energies tend to rapidly increase with the NP size and laser intensity. For the present numerical applications, even small $\eta_{\text{C}}^{R}\left(a,I_{0}\right)$ enable very large cutoff energies, because the coefficient *ωt*_*f*_ in Eq. (5) is large. Assuming an effective PE interaction time *t*_*f*_ ≈ 2*τ*, for the laser parameters used in this study, *ωt*_*f*_ = 120.75.

An unexpected and interesting result derives from the fact that the simulated cutoff energies for direct PEs (“All_Direct”) and rescattered PEs (“All_Net”) are comparable. The direct cutoff energy for 5, 30, and 70 nm NPs are, respectively, 93, 85, and 89 % of the rescattered PE cutoffs for the intensity *I*_0_ and 93, 87, and 84 % for 1.5 *I*_0_. This value is 20 % for strong-field ionization of gaseous atoms, molecules, and dielectric NPs.

## 4 Summary and conclusions

We measured and numerically simulated VMI maps to model strong-field ionization from metal NPs. Our experimental and simulated results scrutinize a complex dynamical interplay of PE emission, propagation, recombination, and rescattering. Augmented by strong plasmonic-field enhancement, a large number of PEs tunnel ionize from metal NPs and result in high PE yields and cutoff energies. We analyzed the size and laser-intensity dependence of PE angular distributions in light of competing contributions from various PE interactions.

We observed that the dipolar shape, imprinted on VMI maps by the incident-laser and induced plasmonic fields, is mostly erased by PE correlation and diffusive rescattering at the NP surface to yield almost isotropic VMI maps. While for gaseous atomic targets, directly emitted PEs acquire no more than about 20 % of the cutoff energy of rescattered PEs [10 *U*_p_(*I*_0_)], we found direct photoemission from metal NPs to yield cutoff energies up to 303 *U*_p_(*I*_0_), reaching between 84 and 93 % of the cutoff energy for rescattered PEs. Due to (exponentially) laser-intensity-dependent PE emission, the effects of residual charges and PE–PE interactions are strongly intensity dependent. This leads to a nonlinear intensity-dependence of the PE yield and cutoff energy scaling with *U*_p_(*I*_0_), contrary to the known linear intensity scaling for gaseous atomic targets.

Our joint experimental and theoretical investigation of a prototypical light-driven nanoplasmonic system supports the use of plasmonic nanostructures towards the development of tunable compact electron and radiation sources for PE and radiation imaging in basic research and for novel photoelectronic detection, catalytic, and light-collecting devices.

## Supplementary Material

Supplementary Material Details

## References

[1] Winterfeldt C., Spielmann C., Gerber G. (2008). Colloquium: optimal control of high-harmonic generation. *Rev. Mod. Phys.*.

[2] Ghimire S., Reis D. A. (2019). High-harmonic generation from solids. *Nat. Phys.*.

[3] Müller M., Kravtsov V., Paarmann A., Raschke M. B., Ernstorfer R. (2016). Nanofocused plasmon-driven sub-10 fs electron point source. *ACS Photonics*.

[4] Yoshida S., Arashida Y., Hirori H. (2021). Terahertz scanning tunneling microscopy for visualizing ultrafast electron motion in nanoscale potential variations. *ACS Photonics*.

[5] Walzl K., Koerting C., Kuppermann A. (1987). Electron-impact spectroscopy of acetaldehyde. *J. Chem. Phys.*.

[6] Venkatraman K., Levin B. D., March K., Rez P., Crozier P. A. (2019). Vibrational spectroscopy at atomic resolution with electron impact scattering. *Nat. Phys.*.

[7] Schötz J., Seiffert L., Maliakkal A. (2021). Onset of charge interaction in strong-field photoemission from nanometric needle tips. *Nanophotonics*.

[8] Kelly K. L., Coronado E., Zhao L. L., Schatz G. C. (2003). The optical properties of metal nanoparticles: the influence of size, shape, and dielectric environment. *J. Phys. Chem. B*.

[9] Wang L., Hasanzadeh Kafshgari M., Meunier M. (2020). Optical properties and applications of plasmonic-metal nanoparticles. *Adv. Funct. Mater.*.

[10] Powell J. A., Li J., Summers A. (2022). Strong-field control of plasmonic properties in core–shell nanoparticles. *ACS Photonics*.

[11] Stockman M. I. (2011). Nanoplasmonics: the physics behind the applications. *Phys. Today*.

[12] Li J., Saydanzad E., Thumm U. (2017). Attosecond time-resolved streaked photoelectron spectroscopy of transition-metal nanospheres. *Phys. Rev. A*.

[13] Link S., El-Sayed M. A. (1999). Size and temperature dependence of the plasmon absorption of colloidal gold nanoparticles. *J. Phys. Chem. B*.

[14] Jain P. K., Huang X., El-Sayed I. H., El-Sayed M. A. (2007). Review of some interesting surface plasmon resonance-enhanced properties of noble metal nanoparticles and their applications to biosystems. *Plasmonics*.

[15] Le Ru E., Etchegoin P. (2008). *Principles of Surface-Enhanced Raman Spectroscopy: And Related Plasmonic Effects*.

[16] Stockman M. I., Kling M. F., Kleineberg U., Krausz F. (2007). Attosecond nanoplasmonic-field microscope. *Nat. Photonics*.

[17] Saydanzad E., Li J., Thumm U. (2022). Strong-field ionization of plasmonic nanoparticles. *Phys. Rev. A*.

[18] Li J., Saydanzad E., Thumm U. (2016). Retrieving plasmonic near-field information: a quantum-mechanical model for streaking photoelectron spectroscopy of gold nanospheres. *Phys. Rev. A*.

[19] Kabashin A., Evans P., Pastkovsky S. (2009). Plasmonic nanorod metamaterials for biosensing. *Nat. Mater.*.

[20] Willets K. A., Van Duyne R. P. (2007). Localized surface plasmon resonance spectroscopy and sensing. *Annu. Rev. Phys. Chem.*.

[21] Saydanzad E., Li J., Thumm U. (2018). Spatiotemporal imaging of plasmonic fields near nanoparticles below the diffraction limit. *Phys. Rev. A*.

[22] Li J., Saydanzad E., Thumm U. (2018). Imaging plasmonic fields with atomic spatiotemporal resolution. *Phys. Rev. Lett.*.

[23] Maurer J., Dimitrovski D., Christensen L., Madsen L. B., Stapelfeldt H. (2012). Molecular-frame 3d photoelectron momentum distributions by tomographic reconstruction. *Phys. Rev. Lett.*.

[24] Lam H. V. S., Wangjam T. N., Kumarappan V. (2022). Alignment dependence of photoelectron angular distributions in the few-photon ionization of molecules by ultraviolet pulses. *Phys. Rev. A*.

[25] Meckel M., Comtois D., Zeidler D. (2008). Laser-induced electron tunneling and diffraction. *Science*.

[26] Powell J. A., Summers A. M., Liu Q. (2019). Interplay of pulse duration, peak intensity, and particle size in laser-driven electron emission from silica nanospheres. *Opt. Express*.

[27] Zherebtsov S., Fennel T., Plenge J. (2011). Controlled near-field enhanced electron acceleration from dielectric nanospheres with intense few-cycle laser fields. *Nat. Phys.*.

[28] Seiffert L., Liu Q., Zherebtsov S. (2017). Attosecond chronoscopy of electron scattering in dielectric nanoparticles. *Nat. Phys.*.

[29] Keldysh L. (1965). Ionization in the field of a strong electromagnetic wave. *Sov. Phys. JETP*.

[30] Paulus G. G., Becker W., Nicklich W., Walther H. (1994). Rescattering effects in above-threshold ionization: a classical model. *J. Phys. B: At., Mol. Opt. Phys.*.

[31] Walker B., Sheehy B., Kulander K. C., DiMauro L. F. (1996). Elastic rescattering in the strong field tunneling limit. *Phys. Rev. Lett.*.

[32] Becker W., Grasbon F., Kopold R., Milošević D., Paulus G., Walther H. (2002). Above-threshold ionization: from classical features to quantum effects. *Adv. At., Mol., Opt. Phys.*.

[33] Becker W., Goreslavski S. P., Milošević D. B., Paulus G. G. (2018). The plateau in above-threshold ionization: the keystone of rescattering physics. *J. Phys. B*.

[34] Ghimire S., DiChiara A. D., Sistrunk E., Agostini P., DiMauro L. F., Reis D. A. (2011). Observation of high-order harmonic generation in a bulk crystal. *Nat. Phys.*.

[35] Ambrosio M. J., Thumm U. (2018). Energy-resolved attosecond interferometric photoemission from Ag(111) and Au(111) surfaces. *Phys. Rev. A*.

[36] Ambrosio M. J., Thumm U. (2019). Spatiotemporal analysis of a final-state shape resonance in interferometric photoemission from Cu(111) surfaces. *Phys. Rev. A*.

[37] Navarrete F., Thumm U. (2020). Two-color-driven enhanced high-order harmonic generation in solids. *Phys. Rev. A*.

[38] Nefedova V. E., Fröhlich S., Navarrete F. (2021). Enhanced extreme ultraviolet high-harmonic generation from chromium-doped magnesium oxide. *Appl. Phys. Lett.*.

[39] Xue L., Liu S., Hang Y. (2021). Unraveling ultrafast photoionization in hexagonal boron nitride. ..

[40] Navarrete F., Ciappina M. F., Thumm U. (2019). Crystal-momentum-resolved contributions to high-order harmonic generation in solids. *Phys. Rev. A*.

[41] Herink G., Solli D. R., Gulde M., Ropers C. (2012). Field-driven photoemission from nanostructures quenches the quiver motion. *Nature*.

[42] Krüger M., Schenk M., Hommelhoff P. (2011). Attosecond control of electrons emitted from a nanoscale metal tip. *Nature*.

[43] Wachter G. (2014). Simulation of condensed matter dynamics in strong femtosecond laser pulses. ..

[44] Krüger M., Schenk M., Hommelhoff P., Wachter G., Lemell C., Burgdörfer J. (2012). Interaction of ultrashort laser pulses with metal nanotips: a model system for strong-field phenomena. *New J. Phys.*.

[45] Wachter G., Lemell C., Burgdörfer J., Schenk M., Krüger M., Hommelhoff P. (2012). Electron rescattering at metal nanotips induced by ultrashort laser pulses. *Phys. Rev. B*.

[46] Kim H., Garg M., Mandal S., Seiffert L., Fennel T., Goulielmakis E. (2023). Attosecond field emission. *Nature*.

[47] Ditmire T., Zweiback J., Yanovsky V., Cowan T., Hays G., Wharton K. (1999). Nuclear fusion from explosions of femtosecond laser-heated deuterium clusters. *Nature*.

[48] Passig J., Irsig R., Truong N. X., Fennel T., Tiggesbäumker J., Meiwes-Broer K. H. (2012). Nanoplasmonic electron acceleration in silver clusters studied by angular-resolved electron spectroscopy. *New J. Phys.*.

[49] Varin C., Peltz C., Brabec T., Fennel T. (2014). Light wave driven electron dynamics in clusters. *Ann. Phys.*.

[50] Lünskens T., Heister P., Thämer M., Walenta C. A., Kartouzian A., Heiz U. (2015). Plasmons in supported size-selected silver nanoclusters. *Phys. Chem. Chem. Phys*..

[51] Wang Z., Camacho Garibay A., Park H. (2020). Universal high-energy photoelectron emission from nanoclusters beyond the atomic limit. *Phys. Rev. Lett.*.

[52] Süßmann F., Kling M. F. (2011). Attosecond nanoplasmonic streaking of localized fields near metal nanospheres. *Phys. Rev. B*.

[53] Seiffert L., Zherebtsov S., Kling M. F., Fennel T. (2022). Strong-field physics with nanospheres. *Adv. Phys. X*.

[54] Corkum P. B. (1993). Plasma perspective on strong field multiphoton ionization. *Phys. Rev. Lett.*.

[55] Powell J. A. (2017). Strong-field driven dynamics of metal and dielectric nanoparticles. ..

[56] Süßmann F., Seiffert L., Zherebtsov S. (2015). Field propagation-induced directionality of carrier-envelope phase-controlled photoemission from nanospheres. *Nat. Commun.*.

[57] Ellis J. L., Hickstein D. D., Xiong W. (2016). Materials properties and solvated electron dynamics of isolated nanoparticles and nanodroplets probed with ultrafast extreme ultraviolet beams. *J. Phys. Chem. Lett.*.

[58] Davino M., Saule T., Helming N., Powell J. A., Trallero-Herrero C. (2022). Characterization of an aerosolized nanoparticle beam beyond the diffraction limit through strong field ionization. *Sci. Rep.*.

[59] Kling N. G., Paul D., Gura A. (2014). Thick-lens velocity-map imaging spectrometer with high resolution for high-energy charged particles. *JINST*.

[60] ..

[61] Rosenberger P., Rupp P., Ali R. (2020). Near-field induced reaction yields from nanoparticle clusters. *ACS Photonics*.

[62] Summers A. M. (2019). Strong-field interactions in atoms and nanosystems: advances in fundamental science and technological capabilities of ultrafast sources. ..

[63] Kumarasinghe C. S., Premaratne M., Bao Q., Agrawal G. P. (2015). Theoretical analysis of hot electron dynamics in nanorods. *Sci. Rep.*.

[64] Narang P., Sundararaman R., Atwater H. A. (2016). Plasmonic hot carrier dynamics in solid-state and chemical systems for energy conversion. *Nanophotonics*.

[65] Jackson J. D. (1999). *Classical Electrodynamics*.

[66] Mie G. (1908). Beiträge zur Optik trüber Medien, speziell kolloidaler Metallösungen. *Ann. Phys.*.

[67] Kuwata H., Tamaru H., Esumi K., Miyano K. (2003). Resonant light scattering from metal nanoparticles: practical analysis beyond the Rayleigh approximation. *Appl. Phys. Lett.*.

[68] Saydanzad E., Li J., Thumm U. (2017). Characterization of induced nanoplasmonic fields in time-resolved photoemission: a classical trajectory approach applied to gold nanospheres. *Phys. Rev. A*.

[69] Haynes W. M. (2014). *CRC Handbook of Chemistry and Physics*.

[70] Fowler R. H., Nordheim L. (1928). Electron emission in intense electric fields. *Proc. R. Soc. London, A*.

[71] Murphy E. L., Good R. (1956). Thermionic emission, field emission, and the transition region. *Phys. Rev.*.

